# Perioperative or adjuvant mFOLFIRINOX for resectable pancreatic cancer (PREOPANC-3): study protocol for a multicenter randomized controlled trial

**DOI:** 10.1186/s12885-023-11141-5

**Published:** 2023-08-07

**Authors:** J. L. van Dam, E. M. M. Verkolf, E. N. Dekker, B. A. Bonsing, S. O. Bratlie, L. A. A. Brosens, O. R. Busch, L. M. J. W. van Driel, C. H. J. van Eijck, S. Feshtali, P. Ghorbani, D. J. A. de Groot, J. W. B. de Groot, B. C. M. Haberkorn, I. H. de Hingh, B. van der Holt, T. M. Karsten, M. B. van der Kolk, K. J. Labori, M. S. L. Liem, O. J. L. Loosveld, I. Q. Molenaar, M. B. Polée, H. C. van Santvoort, J. de Vos – Geelen, M. L. Wumkes, G. van Tienhoven, M. Y. V. Homs, M. G. Besselink, J. W. Wilmink, B. Groot Koerkamp, for the Dutch Pancreatic Cancer Group

**Affiliations:** 1 Department of Surgery, Erasmus MC Cancer Institute, Rotterdam, The Netherlands; 2 Department of Surgery, Leiden University Medical Center, Leiden, The Netherlands; 3 Department of Surgery, Sahlgrenska University Hospital, Gothenburg, Sweden; 4 Department of Pathology, University Medical Center Utrecht, Utrecht University, Utrecht, The Netherlands; 5 Department of Pathology, Radboud UMC, Nijmegen, The Netherlands; 6 Department of Surgery, Amsterdam UMC, Location University of Amsterdam, Amsterdam, The Netherlands; 7 Cancer Center Amsterdam, Amsterdam, The Netherlands; 8 Department of Gastroenterology and Hepatology, Erasmus MC Cancer Institute, Rotterdam, The Netherlands; 9 Department of Radiology, Leiden University Medical Center, Leiden, The Netherlands; 10 Division of Surgery, Department of Clinical Science, Intervention and Technology (CLINTEC), Karolinska Institutet, Stockholm, Sweden; 11 Department of Medical Oncology, University Medical Center Groningen, Groningen, The Netherlands; 12 Department of Medical Oncology, Isala Oncology Center, Zwolle, The Netherlands; 13 Department of Medical Oncology, Maasstad Hospital, Rotterdam, The Netherlands; 14 Department of Surgery, Catharina Hospital, Eindhoven, The Netherlands; 15 Department of Hematology, Erasmus MC Cancer Institute, Rotterdam, The Netherlands; 16 Department of Surgery, OLVG, Amsterdam, The Netherlands; 17 Department of Surgery, Radboud University Medical Center, Nijmegen, The Netherlands; 18 Department of Hepato-Pancreato-Biliary Surgery, Oslo University Hospital, Rikshospitalet and Institute of Clinical Medicine, University of Oslo, Oslo, Norway; 19 Department of Surgery, Medisch Spectrum Twente, Enschede, The Netherlands; 20 Department of Medical Oncology, Amphia Hospital, Breda, The Netherlands; 21 Department of Surgery, Regional Academic Cancer Center Utrecht, St. Antonius Hospital and University Medical Center Utrecht, Utrecht, The Netherlands; 22 Department of Medical Oncology, Medical Center Leeuwarden, Leeuwarden, The Netherlands; 23 Division of Medical Oncology, Department of Internal Medicine, GROW, Maastricht UMC+, Maastricht, the Netherlands; 24 Department of Medical Oncology, Jeroen Bosch Hospital, Den Bosch, The Netherlands; 25 Amsterdam UMC, Department of Radiation Oncology, Location University of Amsterdam, Amsterdam, The Netherlands; 26 Department of Medical Oncology, Erasmus MC Cancer Institute, Rotterdam, The Netherlands; 27 Department of Medical Oncology, Amsterdam UMC, Location University of Amsterdam, Amsterdam, The Netherlands

**Keywords:** Pancreatic cancer, mFOLFIRINOX, Neoadjuvant therapy, Adjuvant therapy, Randomized controlled trial, Overall survival

## Abstract

**Background:**

Surgical resection followed by adjuvant mFOLFIRINOX (5-fluorouracil with leucovorin, irinotecan, and oxaliplatin) is currently the standard of care for patients with resectable pancreatic cancer. The main concern regarding adjuvant chemotherapy is that only half of patients actually receive adjuvant treatment. Neoadjuvant chemotherapy, on the other hand, guarantees early systemic treatment and may increase chemotherapy use and thereby improve overall survival. Furthermore, it may prevent futile surgery in patients with rapidly progressive disease. However, some argue that neoadjuvant therapy delays surgery, which could lead to progression towards unresectable disease and thus offset the potential benefits. Comparison of perioperative (i.e., neoadjuvant and adjuvant) with (only) adjuvant administration of mFOLFIRINOX in a randomized controlled trial (RCT) is needed to determine the optimal approach.

**Methods:**

This multicenter, phase 3, RCT will include 378 patients with resectable pancreatic ductal adenocarcinoma with a WHO performance status of 0 or 1. Patients are recruited from 20 Dutch centers and three centers in Norway and Sweden. Resectable pancreatic cancer is defined as no arterial contact and ≤ 90 degrees venous contact. Patients in the intervention arm are scheduled for 8 cycles of neoadjuvant mFOLFIRINOX followed by surgery and 4 cycles of adjuvant mFOLFIRINOX (2-week cycle of oxaliplatin 85 mg/m^2^, leucovorin 400 mg/m^2^, irinotecan 150 mg/m^2^ at day 1, followed by 46 h continuous infusion of 5-fluorouracil 2400 g/m^2^). Patients in the comparator arm start with surgery followed by 12 cycles of adjuvant mFOLFIRINOX. The primary outcome is overall survival by intention-to-treat. Secondary outcomes include progression-free survival, resection rate, quality of life, adverse events, and surgical complications. To detect a hazard ratio of 0.70 with 80% power, 252 events are needed. The number of events is expected to be reached after the inclusion of 378 patients in 36 months, with analysis planned 18 months after the last patient has been randomized.

**Discussion:**

The multicenter PREOPANC-3 trial compares perioperative mFOLFIRINOX with adjuvant mFOLFIRINOX in patients with resectable pancreatic cancer.

**Trial registration:**

Clinical Trials: NCT04927780. Registered June 16, 2021.

## Introduction

Pancreatic cancer is the fourth leading cause of cancer-related death in Europe and the third in the United States [1, 2]. For patients with resectable pancreatic cancer, surgery followed by adjuvant chemotherapy is currently the standard of care [3, 4]. In 2018, the French-Canadian PRODIGE 24/CCTG PA.6 randomized controlled trial (RCT) showed the superiority of adjuvant mFOLFIRINOX (5-fluorouracil with leucovorin, irinotecan, and oxaliplatin) over adjuvant gemcitabine [5]. Consequently, guidelines recommend adjuvant mFOLFIRINOX for patients with resected pancreatic cancer [3, 4].

The main concern regarding adjuvant chemotherapy is that half of the patients never start adjuvant treatment as a result of surgical complications, clinical deterioration, or early recurrence [6, 7]. Neoadjuvant chemotherapy, on the other hand, guarantees early systemic therapy and may thereby improve overall survival. Furthermore, it may prevent futile surgery in patients with rapidly progressive disease. However, some argue that deterioration during neoadjuvant chemotherapy may preclude surgery and tumors not sensitive to chemotherapy may progress and become unresectable.

Neoadjuvant therapy has been compared with adjuvant therapy in several RCTs for patients with resectable and borderline resectable pancreatic cancer. A recent meta-analysis suggests a survival benefit of neoadjuvant therapy (hazard ratio [HR] 0.66, 95% CI 0.52–0.85; *P* = 0.001) [8]. However, in the subgroup of patients with resectable pancreatic cancer, no statistically significant difference was observed (HR 0.77, 95% CI 0.53–1.12; *P* = 0.18) [8]. Notably, these trials used gemcitabine-based regimens while the current standard of care is adjuvant mFOLFIRINOX [5]. Therefore, an RCT that directly compares neoadjuvant or perioperative to adjuvant administration of mFOLFIRINOX in patients with resectable pancreatic cancer is needed to investigate the optimal approach.

The primary objective of the PREOPANC-3 trial is to investigate whether perioperative mFOLFIRINOX improves overall survival compared with adjuvant mFOLFIRINOX in resectable pancreatic cancer.

## Methods

### Trial design

**Fig. 1:**
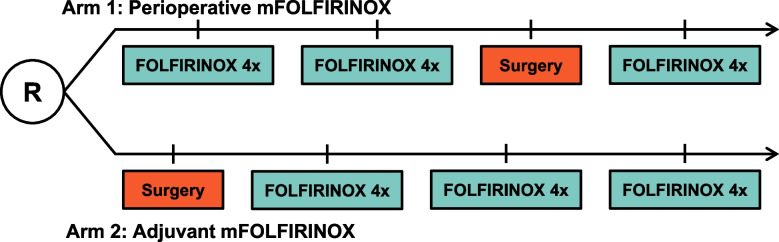
Treatment schedule

The PREOPANC-3 trial is an international, multicenter, phase 3, RCT initiated by the Dutch Pancreatic Cancer Group (DPCG). Patients with resectable pancreatic cancer are randomized to neoadjuvant mFOLFIRINOX followed by surgery and adjuvant mFOLFIRINOX (intervention; arm 1) or to surgery and adjuvant mFOLFIRINOX (comparator; arm 2) (Fig. 1). Randomization is performed centrally in a 1:1 ratio using the minimization technique with World Health Organization (WHO) performance status (0 vs. 1), serum carbohydrate antigen 19–9 (CA 19–9) level (< 300 vs. ≥ 300 U/ml), and center as stratification factors.

### Patients

Eligible patients have cytologically or histologically confirmed non-metastatic, resectable pancreatic ductal adenocarcinoma. Cytological confirmation may include both suspicious (category V) or malignant (category VI) according to the Papanicolaou Society of Cytopathology guidelines [9]. Resectability is determined on computed tomography (CT) imaging. Resectable pancreatic cancer is defined according to the DPCG criteria as no celiac axis, superior mesenteric artery, or common hepatic artery tumor contact and superior mesenteric and/or portal vein tumor contact of 90 degrees or less (Table 1) [10]. The NCCN definition of resectable pancreatic cancer differs from the DPCG definition as it allows for 180 degrees of venous contact [3].

**Table 1:** Dutch Pancreatic Cancer Group criteria defining resectability status at diagnosis

	**Celiac axis**	**Superior mesenteric artery**	**Common hepatic artery**	**Superior mesenteric vein / portal vein**
Resectable (all four required)	no contact	no contact	no contact	≤ 90° contact
Borderline Resectable (minimally one required)	≤ 90° contact	≤ 90° contact	≤ 90° contact	> 90–270° contact and no occlusion
Locally Advanced (minimally one required)	contact > 90°	contact > 90°	contact > 90°	contact > 270° or occlusion

Other inclusion criteria are a WHO performance status of 0 or 1 and adequate hematologic (i.e., hemoglobin ≥ 6 mmol/L [9.7 g/dL]; white blood count ≥ 3.0 × 10^9^/L; platelets ≥ 100 × 10^9^/L) and renal function (i.e., eGFR ≥ 40 ml/min), and written informed consent (Table 2).

**Table 2:** Eligibility criteria

**Inclusion criteria**
• Histological or cytological confirmation (category V: Suspicious or VI: Malignant) of pancreatic ductal adenocarcinoma • Resectable tumor according to DPCG criteria: no arterial contact and venous contact with the superior mesenteric vein or portal vein of 90 degrees or less • No evidence for metastatic disease • WHO performance status of 0 or 1 • Ability to undergo surgery and mFOLFIRINOX chemotherapy • Leucocytes ≥ 3.0 × 10^9^/L • Platelets ≥ 100 × 10^9^/L • Hemoglobin ≥ 6.0 mmol/L • Renal function: eGFR ≥ 40 ml/min • Age ≥ 18 years • Written informed consent
**Exclusion criteria**
• Prior radiotherapy, chemotherapy, or surgery for pancreatic cancer • Prior chemotherapy precluding mFOLFIRINOX • Previous malignancy (excluding non-melanoma skin cancer, pancreatic neuroendocrine tumor (pNET) < 2 cm, and gastrointestinal stromal tumor (GIST) < 2 cm), unless no evidence of disease and diagnosed more than 3 years before diagnosis of pancreatic cancer, or with a life expectancy of more than 5 years from date of inclusion • Pregnancy or lactation • Serious concomitant systemic disorders that would compromise the safety of the patient or his/her ability to complete the study, at the discretion of the investigator

Exclusion criteria are prior radiotherapy, chemotherapy, or surgery for pancreatic cancer. In addition, patients with a previous malignancy (excluding non-melanoma skin cancer, pancreatic neuroendocrine tumor [pNET] < 2 cm, and gastrointestinal stromal tumor [GIST] < 2 cm) are excluded, unless no evidence of disease and diagnosed more than 3 years before diagnosis of pancreatic cancer, or with a life expectancy of more than 5 years from date of inclusion (Table 2).

Patients with biliary obstruction are eligible, but biliary drainage, preferably using an endoscopically placed self-expanding metal stent, must be performed before the initiation of neoadjuvant therapy if bilirubin is above 25 μmol/L (1.5 mg/dL). Last, all patients must be discussed in a multidisciplinary tumor board meeting before inclusion.

### Treatment

#### Both arms: mFOLFIRINOX

The mFOLFIRINOX regimen consists of oxaliplatin 85 mg/m^2^, leucovorin 400 mg/m^2^, irinotecan 150 mg/m^2^, all at day 1, and 5-fluorouracil continuous intravenous infusion 2400 g/m^2^ over 46 h. The dosages are identical to the regimen used in the PRODIGE 24/CCTG PA.6 trial [5]. Testing of the dihydropyrimidine dehydrogenase (DPD) enzyme prior to initiation of mFOLFIRINOX is mandatory as recommended by the European Medicines Agency [11]. In patients with a (partial) deficiency of the DPD enzyme, 5-fluorouracil dose is adjusted or withheld. Primary prophylaxis with granulocyte colony-stimulating factor (G-CSF) after every cycle of mFOLFIRINOX is recommended.

#### Both arms: surgery

Surgical exploration preferably starts with a staging laparoscopy in the same surgical session. If no distant metastases or unresectable disease is found, patients undergo pancreatectomy, which may include pancreatoduodenectomy, distal pancreatectomy, or total pancreatectomy. Both open and minimally-invasive pancreatectomy techniques are allowed.

#### Intervention arm: perioperative mFOLFIRINOX

Patients in the intervention arm start with 4 cycles of neoadjuvant mFOLFIRINOX. After 4 cycles, restaging is performed with a CT scan and tumor markers CA 19–9 and carcinoembryonic antigen (CEA) to rule out progressive disease. If patients have treatment response or stable disease, patients continue with an additional 4 cycles of mFOLFIRINOX. After 8 cycles, restaging with CT scan and tumor markers is repeated. If there is no evidence of disease progression, patients are scheduled for surgical exploration. After resection, patients receive 4 cycles of adjuvant mFOLFIRINOX, starting within 12 weeks after surgery.

During the neoadjuvant phase, if patients do not tolerate the full 8 cycles despite maximal dose reduction but have no signs of progressive disease, earlier surgical exploration is allowed.

#### Comparator arm: adjuvant mFOLFIRINOX

Patients in the comparator arm start with surgical exploration. After resection, patients are scheduled for 12 cycles of adjuvant mFOLFIRINOX. Evaluation of treatment response using CT scan and tumor markers is performed after 4 and 8 cycles. If the patient is unfit to receive adjuvant mFOLFIRINOX, other adjuvant chemotherapy is allowed to the discretion of the treating oncologist.

### Outcomes

The primary outcome of the trial is overall survival defined as the time between randomization and death from any cause. Secondary outcomes include progression-free survival, number of cycles received, dose intensity, resection rate, quality of life, adverse events, and surgical complications. Progression-free survival is defined as the time between randomization and locoregional progressive disease before or during treatment (resulting in irresectability), the occurrence of distant metastases, recurrent pancreatic cancer after surgery or death from any cause. Adverse events are scored according to the NCI Common Terminology Criteria for Adverse Events (CTCAE), version 5 [12]. Surgical complications are defined according to the Clavien-Dindo classification and the International Study Group on Pancreatic Surgery (ISGPS) definitions [13, 14]. In the intervention arm, pathological response and tumor marker response after neoadjuvant therapy are additional outcomes.

#### Follow-up

Patients are followed for a minimum of five years after randomization using a scheduled follow-up scheme. The first two years, follow-up visits take place every three months and patients undergo chest and abdominal CT scan and tumor marker (CA 19–9 and CEA) evaluation every 6 months. Thereafter, follow-up visits take place every 6 months with CT scan and tumor marker evaluation every 12 months. Patients who discontinue study treatment early will be followed for the primary outcome.

### Data collection and management

Study data will be handled confidentially. Each patient will receive an anonymous identification code. To trace data back to an individual patient, a subject identification code list will be used. Each participating site will safeguard the code list for patients included at their site. The handling of personal data will comply with EU General Data Protection Regulation (GDPR) and the Dutch Act on Implementation of the General Data Protection Regulation. Data will be entered on electronic case report forms using the electronic online database ALEA (FormsVision BV, Abcoude, The Netherlands). Data is entered by a small number of trained data managers that collect data from all study sites.

Quality of life is measured during treatment and follow-up using questionnaires. The PAncreatic CAncer Project (PACAP, www.pacap.nl) collects quality of life forms on all patients with pancreatic cancer in the Netherlands.

### Safety and monitoring

Adverse events grade 3 and higher, according to the NCI CTCAE version 5 definitions, that occur within 30 days of last chemotherapy will be recorded [12]. The local investigator will report serious adverse events (SAEs), defined as an adverse event that resulted in death, was life threatening, or required hospitalization, to the sponsor. Suspected Unexpected Serious Adverse Reactions (SUSARs) are reported to the Medical Ethics Committee and competent authority. A monitor team will monitor the study throughout its duration with visits to the study sites. The visits will be conducted to evaluate the progress of the study, ensure accurate data collection, and check compliance with the study protocol. An independent Data Safety Monitoring Board (DSMB) will be installed to evaluate safety data during the study. The DSMB will meet after the first 100 patients have completed treatment and will investigate the occurrence of deaths and adverse events in both treatment arms.

### Statistical methods

Sample size calculation was performed for the primary outcome. The median overall survival in the comparator arm was estimated at 18 months. In order to detect a HR of 0.70 with 80% power and a 5% two-sided significance level, a total of 252 events (deaths) are needed. The number of events is expected to be reached after the inclusion of 378 patients in 36 months, with analysis planned 18 months after the last patient has been randomized. Sample size calculation was performed using East software (East 6 [2020]. Statistical software for the design, simulation and monitoring clinical trials. Cytel Inc., Cambridge MA).

The primary analysis will be performed by intention-to-treat. Survival estimates will be obtained using the Kaplan–Meier method. The stratified log-rank test will be used to test the difference between the treatment arms. Treatment effect will be estimated using a stratified Cox proportional-hazards model. Subgroup analysis according to baseline characteristics will be performed for age, sex, serum CA 19–9 level, tumor location, WHO performance status, tumor size, and venous contact. A p value < 0.05 is considered to indicate statistical significance.

## Discussion

Here, we describe the study protocol for the international, multicenter, phase 3, randomized PREOPANC-3 trial that compares perioperative mFOLFIRINOX with adjuvant mFOLFIRINOX in patients with resectable pancreatic cancer.

Currently, surgical resection followed by adjuvant therapy with mFOLFIRINOX is the standard of care for patients with resectable pancreatic cancer based on the PRODIGE 24/CCTG PA.6 trial [5]. In this trial, the median overall survival in patients treated with adjuvant mFOLFIRINOX was 54.4 months compared with 35.0 months in patients treated with adjuvant gemcitabine. In the long-term analysis, these results were confirmed with a 5-year survival of 43.2% with mFOLFIRINOX compared to 31.4% with gemcitabine [15].

To date, two phase 3 RCTs that compared neoadjuvant with adjuvant therapy for resectable and borderline resectable pancreatic cancer are available. In the Japanese Prep-02/JSAP-05 trial, 364 patients were randomized to neoadjuvant gemcitabine and S-1 chemotherapy, surgery and adjuvant S-1 or to upfront surgery with adjuvant S-1 [16]. Overall survival was better with neoadjuvant therapy (HR 0.72; 95% CI, 0.55 to 0.94; *P* = 0.015). In the Dutch PREOPANC trial, 246 patients were randomized to neoadjuvant gemcitabine-based chemoradiotherapy, surgery, and adjuvant gemcitabine or to upfront surgery with adjuvant gemcitabine [17, 18]. After a median follow-up of 59 months, improved survival was observed with neoadjuvant chemoradiotherapy (HR 0.73; 95% CI, 0.56 to 0.96; *P* = 0.025). Furthermore, a recent meta-analysis of only RCTs including 938 patients found improved survival outcomes with gemcitabine-based neoadjuvant therapy compared with upfront surgery and adjuvant therapy [8]. None of the patients in these trials received adjuvant mFOLFIRINOX as they were designed before publication of the PRODIGE 24/CCTG PA.6 trial. Neoadjuvant mFOLFIRINOX is already the standard of care for patients with borderline resectable pancreatic cancer based on a patient-level meta-analysis and several phase 2 studies [19–21]. However, data regarding the use of neoadjuvant mFOLFIRINOX in patients with resectable pancreatic cancer is limited. The phase 2 SWOG S1505 trial that compared perioperative mFOLFIRINOX (6 cycles neoadjuvant; 6 cycles adjuvant) with perioperative gemcitabine/nab-paclitaxel (3 cycles neoadjuvant; 3 cycles adjuvant) in 102 patients with resectable pancreatic cancer found a median overall survival of 23.2 months and a resection rate of 73% in patients treated with mFOLFIRINOX [22]. In the three-arm, phase 2, PANACHE01-PRODIGE48 trial, 153 patients were randomized to 4 cycles of neoadjuvant mFOLFIRINOX, 4 cycles of neoadjuvant FOLFOX or to upfront surgery with adjuvant chemotherapy [23]. Patients in the neoadjuvant arms received 8 cycles of adjuvant chemotherapy. One year overall survival was 84.1% with neoadjuvant mFOLFIRINOX, 71.8% with neoadjuvant FOLFOX, and 80.8% with upfront surgery. Resection rates were 74%, 68%, and 81%, respectively. The largest retrospective series of neoadjuvant mFOLFIRINOX in patients with resectable pancreatic cancer included 346 patients from 5 centers in the United States and the Netherlands and found a median overall survival of 31.2 months and a resection rate of 70.5% [24].

Comparison of survival outcomes of neoadjuvant and adjuvant trials is difficult, if not impossible. Adjuvant trials include only patients who have undergone resection. These patients successfully underwent pancreatectomy and recovered adequately to be considered for adjuvant therapy. In addition, adjuvant trials require a recent CT scan and some trials had upper limits for post-resection CA 19–9. In contrast, neoadjuvant trials include patients with resectable pancreatic cancer on imaging. As a result, neoadjuvant trials include patients that are found to have metastases or unresectable disease at surgical exploration, patients who died from surgery, and patients with surgical complications or clinical deterioration precluding adjuvant therapy. A recent meta-analysis of 7 RCTs demonstrated that even in the upfront surgery arm 1 in 5 patients underwent no resection, mostly due to metastatic disease found during surgery [8].

In the PREOPANC-3 trial, patients in the intervention arm are scheduled for 4 cycles of adjuvant mFOLFIRINOX after 8 cycles of neoadjuvant mFOLFIRINOX and resection. The exact benefit of adjuvant chemotherapy after neoadjuvant FOLFIRINOX and resection is unclear. An international cohort study that investigated the value of adjuvant therapy after neoadjuvant mFOLFIRINOX found improved survival only in the subgroup of patients with pathology proven node-positive disease [25]. We chose 12 cycles of mFOLFIRINOX in both arms to balance the total number of scheduled cycles between the treatment arms.

Radiotherapy is not part of the neoadjuvant treatment in the intervention arm. Currently, there is insufficient evidence that neoadjuvant (chemo)radiotherapy improves overall survival when scheduled in combination with neoadjuvant mFOLFIRINOX. A meta-analysis including 512 patients with resectable and borderline resectable pancreatic cancer treated with radiotherapy after FOLFIRINOX found higher R0 resection rates without improved overall survival [26]. In addition, a propensity matched analysis including 300 patients with borderline resectable pancreatic cancer treated with and without radiotherapy found similar overall survival [27]. The recent phase 2 ALLIANCE A021501 RCT also found no survival benefit of mFOLFIRINOX followed by stereotactic body radiotherapy (SBRT) compared with mFOLFIRINOX alone in patients with borderline resectable pancreatic cancer [21].

Accrual is challenging in RCTs that compare perioperative chemotherapy to upfront surgery in patients with pancreatic cancer. In a recent meta-analysis, only three of the seven trials were able to complete accrual [8]. The DPCG has demonstrated with the PREOPANC and PREOPANC-2 trials that it can reach timely and complete accrual [17, 28].

Currently, two other RCTs compare perioperative with adjuvant administration of mFOLFIRINOX in patients with resectable pancreatic cancer. In the Scandinavian NorPACT-1 trial (NCT02919787), 140 patients were randomized between 2017 and 2021 to 4 cycles of neoadjuvant mFOLFIRINOX followed by surgery and 8 cycles of adjuvant mFOLFIRINOX or to surgery and 12 cycles of adjuvant mFOLFIRINOX. Results are expected by the end of 2022. In the United States, the ALLIANCE A021806 trial (NCT04340141) randomizes 352 patients to 8 cycles of neoadjuvant mFOLFIRINOX followed by surgery and 4 cycles of adjuvant mFOLFIRINOX or to surgery and 12 cycles of adjuvant mFOLFIRINOX.

In conclusion, the PREOPANC-3 trial is designed to compare perioperative mFOLFIRINOX with adjuvant mFOLFIRINOX in patients with resectable pancreatic cancer.

### Trial status

The PREOPANC-3 trial opened for accrual on August 24, 2021 and the first patient was randomized on September 7, 2021. As of January 1, 2023, 16 of the 23 planned hospitals are open for inclusion and 100 patients have been randomized.

## Data Availability

Data sharing not applicable to this article as no datasets were generated or analyzed during the current study.

## Abbreviations

CA 19–9: Carbohydrate antigen 19–9
CEA: Carcinoembryonic antigen
CT: Computed tomography
DPCG: Dutch Pancreatic Cancer Group
DPD: Dihydropyrimidine dehydrogenase
DSMB: Data Safety Monitoring Board
eGFR: Estimated glomerular filtration rate
mFOLFIRINOX: 5-Fluorouracil with leucovorin, irinotecan, and oxaliplatin
G-CSF: Granulocyte colony stimulating factor
GDPR: General Data Protection Regulation
GIST: Gastrointestinal stromal tumor
HR: Hazard ratio
ISGPS: International Study Group of Pancreatic Surgery
NCCN: National Comprehensive Cancer Network
pNET: Pancreatic neuroendocrine tumor
RECIST: Response evaluation criteria in solid tumors
SAE: Serious adverse event
SUSAR: Suspected Unexpected Serious Adverse Reactions
WHO: World Health Organization
